# Immunoglobulin G N-glycan, inflammation and type 2 diabetes in East Asian and European populations: a Mendelian randomization study

**DOI:** 10.1186/s10020-022-00543-z

**Published:** 2022-09-14

**Authors:** Biyan Wang, Di Liu, Manshu Song, Wei Wang, Bo Guo, Youxin Wang

**Affiliations:** 1 Beijing Municipal Key Laboratory of Clinical Epidemiology, School of Public Health, Capital Medical University, 10 Xitoutiao, Beijing, 100069 China; 2 Centre for Biomedical Information Technology, Shenzhen Institutes of Advanced Technology, Chinese Academy of Sciences, Shenzhen, Guangdong China; 3 School of Medical and Health Sciences, Edith Cowan University, Perth, WA 6027 Australia; 4 Department of Hematology, The Second Medical Centre and National Clinical Research Center for Geriatric Diseases, Chinese PLA General Hospital, 28 Fuxing Rd, Beijing, 100853 China

**Keywords:** Type 2 diabetes, Immunoglobulin G, N-glycans, Inflammation, Mendelian randomization

## Abstract

**Background:**

Immunoglobulin G (IgG) N-glycans have been shown to be associated with the risk of type 2 diabetes (T2D) and its risk factors. However, whether these associations reflect causal effects remain unclear. Furthermore, the associations of IgG N-glycans and inflammation are not fully understood.

**Methods:**

We examined the causal associations of IgG N-glycans with inflammation (C-reactive protein (CRP) and fibrinogen) and T2D using two-sample Mendelian randomization (MR) analysis in East Asian and European populations. Genetic variants from IgG N-glycan quantitative trait loci (QTL) data were used as instrumental variables. Two-sample MR was conducted for IgG N-glycans with inflammation (75,391 and 18,348 participants of CRP and fibrinogen in the East Asian population, 204,402 participants of CRP in the European population) and T2D risk (77,418 cases and 356,122 controls of East Asian ancestry, 81,412 cases and 370,832 controls of European ancestry).

**Results:**

After correcting for multiple testing, in the East Asian population, genetically determined IgG N-glycans were associated with a higher risk of T2D, the odds ratios (ORs) were 1.009 for T2D per 1- standard deviation (SD) higher GP5, 95% CI = 1.003–1.015; *P* = 0.0019; and 1.013 for T2D per 1-SD higher GP13, 95% CI = 1.006–1.021; *P* = 0.0005. In the European population, genetically determined decreased GP9 was associated with T2D (OR = 0.899 per 1-SD lower GP9, 95% CI: 0.845–0.957). In addition, there was suggestive evidence that genetically determined IgG N-glycans were associated with CRP in both East Asian and European populations after correcting for multiple testing, but no associations were found between IgG N-glycans and fibrinogen. There was limited evidence of heterogeneity and pleiotropy bias.

**Conclusions:**

Our results provided novel genetic evidence that IgG N-glycans are causally associated with T2D.

**Supplementary Information:**

The online version contains supplementary material available at 10.1186/s10020-022-00543-z.

## Background

Type 2 diabetes (T2D) is a chronic disease characterized by relative insulin deficiency and peripheral insulin resistance with a high societal burden (Chatterjee et al. 2017; Khan et al. 2020; Liang et al. 2020). It is estimated that over 642 million T2D patients by 2030 (Chen et al. 2011). Although many environmental and genetic risk factors have been identified, the underlying mechanisms of different polygenic multifactorial chronic complex disorders like T2D remain uncertain (Tremblay and Hamet 2019).

Glycosylation is the most frequent posttranslational modification, constituting the composition of proteins, modulating their function and participating in many key biological processes, including protein folding, molecular trafficking and clearance, cell adhesion and immune regulation (Krištić and Lauc 2017; Kronimus et al. 2019). Immunoglobulin G (IgG) represents a highly abundant protein and constitutes an important part of humoral immune response (Nimmerjahn and Ravetch 2008). N-glycans are linked to conserved asparagine-297 in the Fc part of IgG, and switch its function between pro- and anti-inflammatory (Kronimus et al. 2019; Yamaguchi and Barb 2020). C-reactive protein (CRP) and fibrinogen can be considered as well-proven clinical markers of systemic inflammation, which are synthesized by hepatocytes against inflammation (Dalmon et al. 1993; Pepys and Hirschfield 2003). Previous studies suggest that alterations of IgG N-glycans can mediate inflammatory responses, and thus are associated with T2D risk (Novokmet et al. 2014; Plomp et al. 2017). A number of observational studies have also demonstrated that aberrant IgG N-glycans affect the pathogenesis of several chronic inflammatory diseases (Ercan et al. 2010; Jin et al. 2013; Trbojević Akmačić et al. 2015; Liu et al. 2018b; Pavić et al. 2018).

Limited data available from observational studies suggested that IgG N-glycan changes have been linked to clinical risk factors of T2D, such as age (Krištić et al. 2014), BMI (Nikolac Perkovic et al. 2014; Greto et al. 2021), and dyslipidemia (Liu et al. 2018a). Furthermore, IgG N-glycans have been identified to be correlated with T2D in several case–control and prospective studies (Lemmers et al. 2017; Dotz et al. 2018; Li et al. 2019; Wittenbecher et al. 2020). Although there is a growing consensus that the role of IgG N-glycosylation is involved in the development of T2D, it remains unclear whether the associations of IgG N-glycans and T2D are causal or a proxy for unmeasured confounding factors, and the population-scale evidence for the effects of IgG N-glycans on inflammation is scarce. Understanding the specific associations of IgG N-glycosylation and T2D would help to reveal the underlying inflammatory pathophysiological processes and drug target of T2D. Consequently, further study is needed to investigate the causality between the alterations of IgG N-glycosylation and T2D.

Mendelian randomization (MR) studies, which use genetic variants as instrumental variables for exposure, can further support causality due to their randomly allocated nature and circumvent confounding and reverse causation (Emdin et al. 2017). None of previous study on causality of IgG N-glycans and T2D used the MR design so far. In this study, given the ethnic-specific differences of IgG N-glycans (Gebrehiwot et al. 2018; Štambuk et al. 2020), we aimed to estimate the effects of genetically predicted IgG N-glycans on T2D risk using two-sample MR in East Asian and European populations, respectively. Second, we aimed to examine the potential associations of genetically predicted IgG N-glycans with inflammation (including CRP and fibrinogen).

## Methods

### Study design and data source

**Fig. 1:**
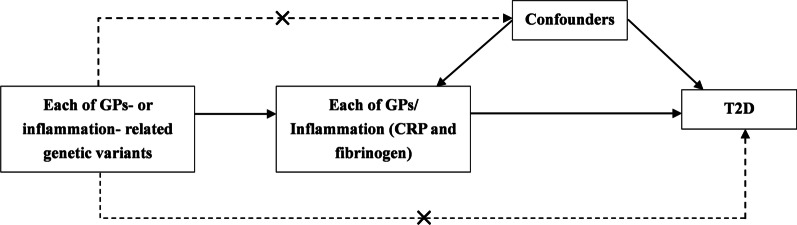
Design of the current two-sample Mendelian randomization analysis. *GP* glycan peak, *CRP* C-reactive protein, *T2D* type 2 diabetes

We used two-sample MR to examine the associations of IgG N-glycans with T2D risk, and IgG N-glycans with inflammation (including CRP and fibrinogen) in East Asian and European populations (Fig. 1). The two-sample MR design is under the assumption that the genetic variants are associated with exposure, but not with confounders. Besides, the genetic variants affect risk of outcome only through exposure and not through any alternative pathways.

In the East Asian population, information on genetic variants associated with IgG N-glycan was collected from IgG N-glycan quantitative trait loci (IgG N-glycan-QTL) summarized data (Liu et al. 2018a, 2022). This genome-wide association study (GWAS) involved 536 individuals from a cross-sectional study, and 7,108,659 single nucleotide polymorphisms (SNPs) of each IgG N-glycan (24 glycan peaks) (excluding SNP with call rate < 99%, minor allele frequency < 0.01 and imputation quality ratio < 0.7) (Liu et al. 2022). The IgG N-glycans were quantified using ultra-performance liquid chromatography (UPLC) and 24 glycan peaks (GPs) were separated from IgG N-glycans. The detail structures of each glycan peak are descripted in Additional file 2: Table S1. Genotyping was performed on Illumina Omni Zhonghua chips (Illumina, San Diego, CA, USA) in this GWAS (Liu et al. 2020). Summary statistics data on associations of genetic variants with CRP and fibrinogen were accessed through a published large-scale GWAS by Biobank Japan, which included 75,391 and 18,348 participants respectively (Kanai et al. 2018). Data on association of genetic variants with T2D were obtained from a published GWAS meta-analysis by of 77,418 cases and 356,122 controls (Spracklen et al. 2020). In the European population, summary statistics data on associations of genetic variants with IgG N-glycan were accessed through a recently published GWAS (Klarić et al. 2020). The IgG N-glycans were also detected by UPLC and separated to 24 GPs. We also obtained association summary statistics with CRP and T2D from respective GWAS studies (Ligthart et al. 2018; Mahajan et al. 2018). GWASs included in the current study are described in detail in Table 1.

**Table 1:** Characteristics of the GWAS used in this study

Phenotype	Sample size	Ancestry	Citation	PMID
IgG N-glycans	536	East Asian	Liu et al. (2022)	35545292
CRP	75,391	East Asian	Kanai et al. (2018) http://jenger.riken.jp/en/result/	29403010
Fibrinogen	18,348	East Asian	Kanai et al. (2018) http://jenger.riken.jp/en/result/	29403010
T2D	77,418 cases and 356,122 controls	East Asian	Spracklen et al. (2020)	32499647
IgG N-glycans	8090	European	Klarić et al. (2020)	32128391
CRP	204,402	European	Ligthart et al. (2018)	30388399
T2D	81,412 cases and 370,832 controls	European	Mahajan et al. (2018)	29632382

*GWAS* genome-wide association study, *IgG* Immunoglobulin G, *CRP* C-reactive protein, *T2D* type 2 diabetes

### Genetic instruments for IgG N-glycans

Independent SNPs (low linkage disequilibrium (LD), R^2^ < 0.001) associated with IgG N-glycans were identified as instrumental variables (IVs). The selected SNPs were independent, namely, not in linkage disequilibrium (R^2^ < 0.001). Independently associated SNPs (*P* < 5 × 10^–8^) with IgG N-glycans were selected in European ancestry. Because few genetic variants were available, referring previous studies (Savage et al. 2018; Dong et al. 2021), a relatively relaxed threshold (*P* < 1 × 10^–5^) was used to select SNPs in the East Asian population. Then, the *F*_statistic (beta^2^/se^2^) was applied to evaluate the strength for each SNP, and SNPs with less statistical power (*F*-statistics < 10) were removed to avoid weak IV bias.

### Statistical analysis

First, two-sample MR analyses were performed to disentangle the potential associations of IgG N-glycans and T2D. For MR analyses, conventional inverse variance weighting (IVW) (Burgess et al. 2013) and three sensitivity analyses including MR-Egger regression (Bowden et al. 2015), weighted median (WM) (Bowden et al. 2016) and penalized weighted median (PWM) were utilized to compute robust causal estimates on outcome of exposures (Liu et al. 2021). When only 2 SNPs were available, the IVW method was used. Sensitivity analysis was used when more than 2 SNPs are available. Heterogeneity of individual genetic variants effect was estimated by Cochran’s Q test (Bowden et al. 2018). Multiplicative random effects IVW models are used when heterogeneity exist. MR-Egger regression analysis can also assess the directional pleiotropy based on its intercepts (Burgess and Thompson 2017). Only 2 SNPs were available, thus we searched each IV in the PhenoScanner v2 to determine potential pleiotropy (Kamat et al. 2019) (Additional file 1: Table S2). In addition, we also provided scatter plots and leave-one-out plots for further interpretation. Leave-one-out analyses were performed to test the influence of outlying or genetic variants pleiotropy (Burgess and Thompson 2017). All results are presented as odds ratios (OR) with their 95% confidence interval (CI) of outcomes per genetically predicted increase in each exposure factor. To take into account multiple testing, Bonferroni corrected *P* value threshold for 24/17 exposures (*P* < 0.002/0.003) was used to indicate statistical significance. *P* < 0.05 but above the Bonferroni corrected significance threshold was considered as suggestive evidence for a potential association. All analyses were performed with R (version 4.0.2) with the “TwoSampleMR” and “MendelianRandomization” packages.

## Results

### Associations of IgG N-glycans with T2D risk

**Fig. 2:**
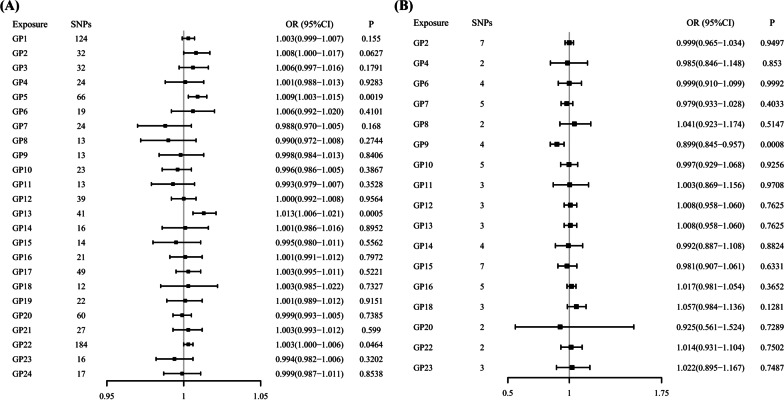
Odds ratios (OR) and 95% confidence intervals (CI) of the associations between genetically determined IgG N-glycans and T2D. **A** in the East Asian population. **B** in the European population. *GP* glycan peak, *T2D* type 2 diabetes, *SNP* single nucleotide polymorphism

**Fig. 3:**
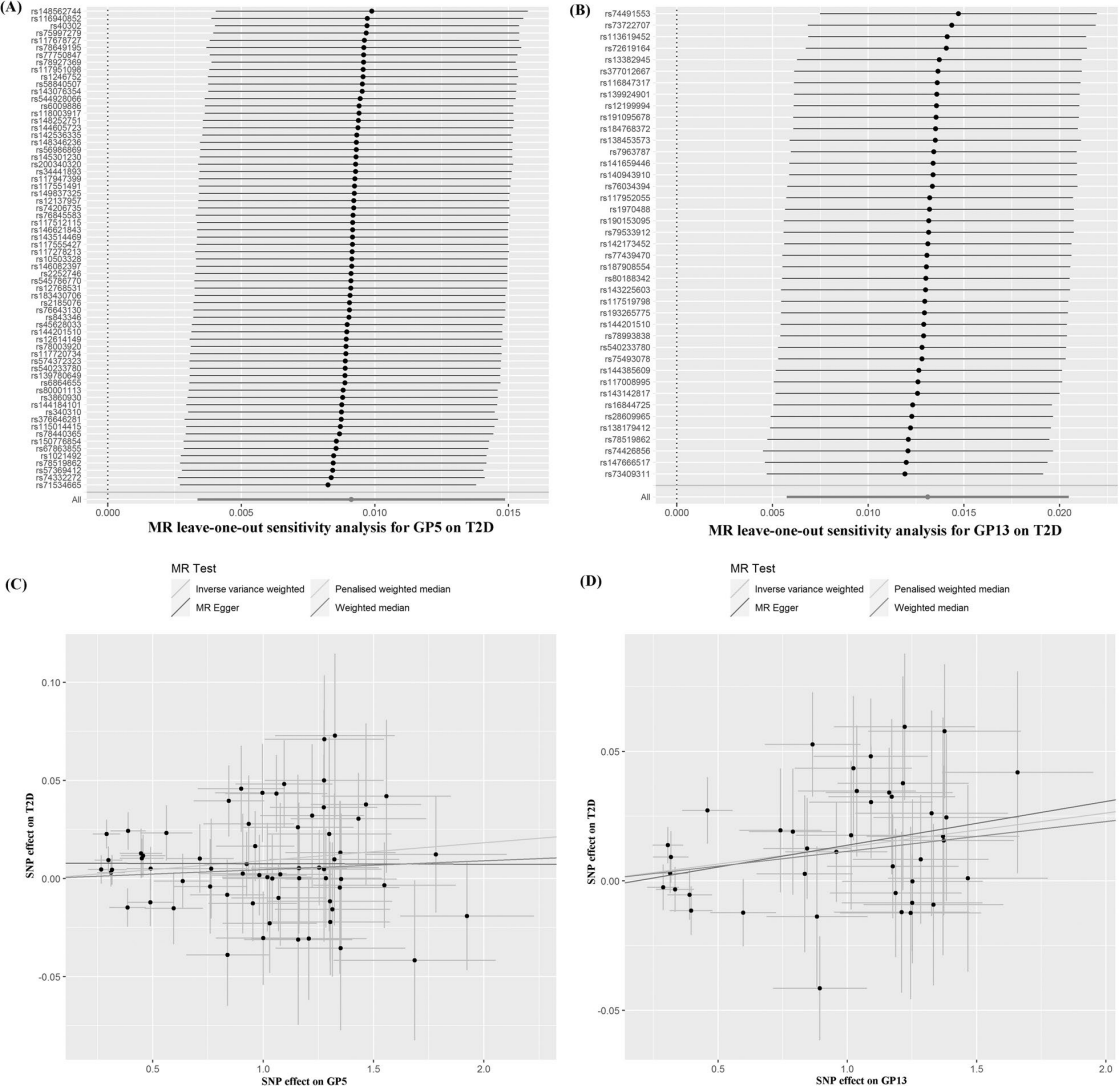
Leave-one-out plots and scatter plots of IgG N-glycans on T2D in the East Asian population. **A** Leave-one-out plots of GP5 on T2D. **B** Leave-one-out plots of GP13 on T2D. **C** Scatter plots of GP5 on T2D. **D** Scatter plots of GP13 on T2D. Leave-one-out plots show the estimate of GP5/GP13 on T2D after the corresponding SNP was excluded. Scatter plots show the per-allele association with T2D plotted against the per-allele association with one standard deviation of GP5/GP13 (vertical and horizontal black lines presenting the 95% CI for each SNP), with the slope of each line corresponding to estimate the effect per method. *SNP* single nucleotide polymorphism, *GP* glycan peak, *T2D* type 2 diabetes, *IgG* immunoglobulin G

The summary genetic association data of each GP are reported in the Additional file 1: Tables S3 and S4. The *F*-statistics for all SNPs are > 10 (Additional file 1: Tables S3 and S4), suggesting that the selected SNPs have sufficiently strong effects as IVs and unlikely have weak instrument bias. In the East Asian population, MR results showed positive effects of genetically determined GP5 and GP13 on T2D after multiple testing (IVW OR, 1.009 for T2D per 1-SD higher GP5, 95% CI = 1.003–1.015; *P* = 0.0019; 1.013 for T2D per 1-SD higher GP13, 95% CI = 1.006–1.021; *P* = 0.0005; Figs. 2, 3 and Table 2). The results of genetically determined GP13 with T2D are remain robust in three sensitivity analysis (WM OR_GP13_ = 1.012, 95% CI = 1.001–1.023, PWM OR_GP13_ = 1.013, 95% CI = 1.006–1.021, MR-Egger OR_GP13_ = 1.003, 95% CI = 1.000–1.006). However, no significant effects of genetically predicted GP5 and GP22 on T2D were observed in WM and PWM estimates (Table 2). The MR analyses showed that there was no pleiotropy bias and heterogeneity in 21 genetically instrumented IgG N-glycans (all *P*s > 0.05), except GP5, GP7, and GP17 (Table 2). Additionally, the leave-one-out analysis showed no marked difference in causal estimations of GP5 and GP13 on T2D in the conditions that anyone SNP was excluded, suggesting that the inverse associations were not substantially driven by any individual SNP (Fig. 3).

**Table 2:** MR estimates of IgG glycans with T2D in the East Asian population

Exposure	SNPs	IVW		WM		PWM		MR-Egger		*P*_pleiotropy_	*P*_heterogeneity_
		OR (95%CI)	*P*	OR (95%CI)	*P*	OR (95%CI)	*P*	OR (95%CI)	*P*		
GP1	124	1.003(0.999–1.007)	0.1550	1.000(0.994–1.006)	0.9993	1.000(0.994–1.005)	0.9704	0.998(0.990–1.006)	0.5792	0.1423	0.1989
GP2	32	1.008(1.000–1.017)	0.0627	1.013(1.001–1.026)	0.0401	1.013(1.001–1.026)	0.0300	1.009(0.993–1.025)	0.2740	0.9198	0.6452
GP3	32	1.006(0.997–1.016)	0.1791	1.007(0.995–1.019)	0.2574	1.009(0.997–1.021)	0.1446	0.996(0.979–1.014)	0.6859	0.2018	0.1330
GP4	24	1.001(0.988–1.013)	0.9283	0.998(0.982–1.014)	0.7695	0.997(0.981–1.013)	0.7380	1.005(0.981–1.029)	0.7126	0.7046	0.1766
GP5	66	1.009(1.003–1.015)	0.0019	1.005(0.996–1.013)	0.3010	1.004(0.997–1.012)	0.2684	1.000(0.989–1.010)	0.9593	0.0446	0.2855
GP6	19	1.006(0.992–1.020)	0.4101	1.004(0.985–1.022)	0.6993	1.003(0.985–1.022)	0.7091	1.003(0.979–1.028)	0.7973	0.8061	0.2320
GP7	24	0.988(0.970–1.005)	0.1680	0.995(0.981–1.009)	0.4491	0.995(0.981–1.009)	0.5037	0.984(0.948–1.021)	0.3885	0.8055	< 0.0001
GP8	13	0.990(0.972–1.008)	0.2744	0.987(0.967–1.008)	0.2174	0.981(0.960–1.001)	0.0663	0.976(0.931–1.023)	0.3313	0.5336	0.0642
GP9	13	0.998(0.984–1.013)	0.8406	1.003(0.983–1.022)	0.7996	1.003(0.984–1.022)	0.7905	0.988(0.952–1.025)	0.5315	0.5477	0.2248
GP10	23	0.996(0.986–1.005)	0.3867	0.997(0.981–1.013)	0.6886	0.997(0.981–1.012)	0.6393	1.008(0.986–1.030)	0.4867	0.2328	0.5558
GP11	13	0.993(0.979–1.007)	0.3528	1.000(0.981–1.020)	0.9860	1.000(0.981–1.020)	0.9789	0.996(0.965–1.028)	0.7961	0.8698	0.5027
GP12	39	1.000(0.992–1.008)	0.9564	1.001(0.990–1.013)	0.8344	1.001(0.990–1.012)	0.8446	1.007(0.993–1.022)	0.3441	0.2724	0.3584
GP13	41	1.013(1.006–1.021)	0.0005	1.012(1.001–1.023)	0.0377	1.012(1.001–1.023)	0.0371	1.017(1.003–1.031)	0.0227	0.5362	0.7358
GP14	16	1.001(0.986–1.016)	0.8952	1.002(0.982–1.022)	0.8563	1.002(0.982–1.022)	0.8580	0.998(0.964–1.034)	0.9310	0.8748	0.1536
GP15	14	0.995(0.980–1.011)	0.5562	1.006(0.986–1.025)	0.5835	1.007(0.987–1.026)	0.5174	0.977(0.939–1.015)	0.2575	0.3193	0.4690
GP16	21	1.001(0.991–1.012)	0.7972	1.000(0.985–1.014)	0.9610	1.000(0.985–1.015)	0.9615	1.001(0.974–1.029)	0.9269	0.9977	0.0111
GP17	49	1.003(0.995–1.011)	0.5221	1.001(0.991–1.011)	0.8529	1.001(0.991–1.011)	0.8459	1.011(0.995–1.026)	0.1783	0.2327	0.1484
GP18	12	1.003(0.985–1.022)	0.7327	1.015(0.992–1.038)	0.2019	1.017(0.995–1.040)	0.1300	0.986(0.939–1.035)	0.5792	0.4608	0.0671
GP19	22	1.001(0.989–1.012)	0.9151	1.004(0.990–1.018)	0.5898	1.004(0.990–1.018)	0.5978	1.011(0.988–1.035)	0.3525	0.3129	0.5478
GP20	60	0.999(0.993–1.005)	0.7385	0.996(0.988–1.004)	0.3332	0.996(0.987–1.004)	0.3304	1.004(0.991–1.018)	0.5040	0.3621	0.3163
GP21	27	1.003(0.993–1.012)	0.5990	0.998(0.985–1.012)	0.8107	0.998(0.985–1.012)	0.8143	1.001(0.981–1.021)	0.9496	0.8277	0.1124
GP22	184	1.003(1.000–1.006)	0.0464	1.003(0.999–1.008)	0.1216	1.003(0.999–1.008)	0.1326	1.007(1.000–1.015)	0.0441	0.1875	0.3638
GP23	16	0.994(0.982–1.006)	0.3202	0.999(0.982–1.016)	0.9474	1.001(0.985–1.017)	0.9256	0.992(0.963–1.021)	0.5822	0.8687	0.4984
GP24	17	0.999(0.987–1.011)	0.8538	1.001(0.985–1.018)	0.8899	1.001(0.985–1.018)	0.8887	1.012(0.978–1.047)	0.5068	0.4414	0.4984

*MR* Mendelian randomization, *T2D* type 2 diabetes, *IVW* inverse variance weighting, *WM* weighted median, *PWM* penalized weighted median, *OR* odds ratio, *CI* confidence interval, *SNP* single-nucleotide polymorphism, *GP* glycan peak, *P*_heterogeneity_ is the *P*-value of Cochrane’s *Q* value in heterogeneity test by performing Inverse variance weighted method; *P*_pleiotropy_ is the *P*-value of MR-Egger intercept

**Fig. 4:**
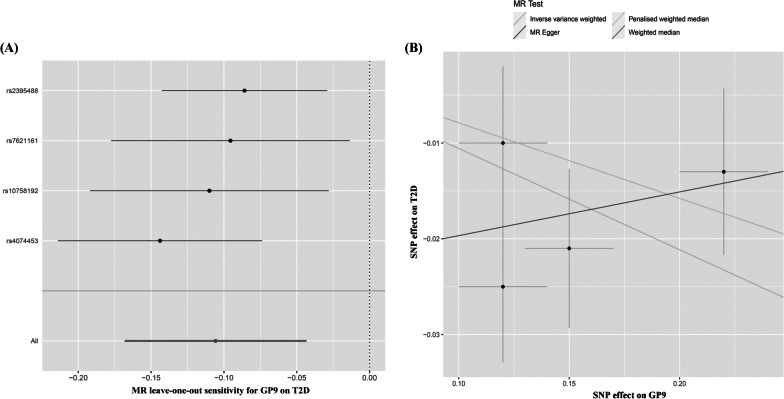
Leave-one-out plots and scatter plots of IgG N-glycans of GP9 on T2D in the European population. Leave-one-out plots show the estimate of GP9 on T2D after the corresponding SNP was excluded. Scatter plots show the per-allele association with T2D plotted against the per-allele association with one standard deviation of GP9 (vertical and horizontal black lines presenting the 95% CI for each SNP), with the slope of each line corresponding to estimate the effect per method. SNP, single nucleotide polymorphism; GP, glycan peak; T2D, type 2 diabetes; IgG, immunoglobulin G

In the European population, genetically determined GP9 is associated with a higher risk of T2D after multiple testing (IVW OR, 0.899 for T2D per 1-SD lower GP9, 95% CI = 0.845–0.957; *P* = 0.0008; Figs. 2, 4 and Table 3). The results of genetically determined GP9 with T2D are remain robust in WM and PWM sensitivity analysis (WM OR_GP9_ = 0.924, 95% CI = 0.866–0.985, PWM OR_GP9_ = 0.924, 95% CI = 0.868–0.984). And the results of IVW are reliable from the results of directional pleiotropy of GP9 on T2D (Table 3). However, no significant effects of genetically predicted GP5 and GP13 on T2D were observed in the European population. The MR-Egger method detected no evidence of directional pleiotropy among each GP for T2D (all *P*s > 0.05, Table 3). IVW results using multiplicative random-effects models are presented when heterogeneity exist. The results of leave-one-out sensitivity analysis support that no single SNP drove the overall association between GP9 and T2D (Fig. 4).

**Table 3:** MR estimates of IgG glycans with T2D in the European population

Exposure	SNPs	IVW		WM		PWM		MR-Egger		*P*_pleiotropy_	*P*_heterogeneity_
		OR (95%CI)	*P*	OR (95%CI)	*P*	OR (95%CI)	*P*	OR (95%CI)	*P*		
GP2	7	0.999(0.965–1.034)	0.9497	1.009(0.971–1.048)	0.6515	1.010(0.969–1.052)	0.6251	1.042(0.947–1.146)	0.4365	0.3931	0.2717
GP4	2	0.985(0.846–1.148)	0.8530	–							0.0881
GP6	4	0.999(0.910–1.099)	0.9992	0.966(0.896–1.042)	0.3766	0.956(0.884–1.034)	0.2642	1.049(0.459–2.856)	0.8005	0.7990	0.0361
GP7	5	0.979(0.933–1.028)	0.4033	0.993(0.941–1.049)	0.8131	0.994(0.941–1.049)	0.8215	1.055(0.953–1.168)	0.2080	0.2080	0.3010
GP8	2	1.041(0.923–1.174)	0.5147	–							0.1993
GP9	4	0.899(0.845–0.957)	0.0008	0.924(0.866–0.985)	0.0198	0.924(0.868–0.984)	0.0198	1.046(0.853–1.284)	0.7046	0.2723	0.2293
GP10	5	0.997(0.929–1.068)	0.9256	0.963(0.910–1.018)	0.1831	0.951(0.902–1.002)	0.0617	1.033(0.820–1.301)	0.8003	0.7669	0.0137
GP11	3	1.003(0.869–1.156)	0.9708	0.938(0.867–1.014)	0.1077	0.937(0.869–1.012)	0.0957	0.925(0.476–1.799)	0.8455	0.8455	0.0035
GP12	3	1.008(0.958–1.060)	0.7625	1.009(0.955–1.067)	0.7363	1.009(0.955–1.067)	0.7361	1.046(0.903–1.213)	0.6546	0.6890	0.8679
GP13	3	1.008(0.958–1.060)	0.7625	1.009(0.956–1.066)	0.7324	1.009(0.954–1.067)	0.7405	1.046(0.903–1.212)	0.6546	0.6890	0.8679
GP14	4	0.992(0.887–1.108)	0.8824	0.928(0.905–1.043)	0.4290	0.928(0.853–1.009)	0.0838	0.936(0.538–1.627)	0.8365	0.8524	0.0034
GP15	7	0.981(0.907–1.061)	0.6331	0.966(0.900–1.035)	0.3258	0.951(0.889–1.016)	0.1361	0.939(0.551–1.603)	0.8283	0.8791	0.0053
GP16	5	1.017(0.981–1.054)	0.3652	1.032(1.003–1.062)	0.0304	1.033(1.004–1.063)	0.0248	1.099(1.028–1.175)	0.0691	0.0910	0.0655
GP18	3	1.057(0.984–1.136)	0.1281	1.064(1.000–1.132)	0.0499	1.064(1.000–1.132)	0.0490	1.216(0.684–2.162)	0.6246	0.7125	0.1389
GP20	2	0.925(0.561–1.524)	0.7289	–							< 0.0001
GP22	2	1.014(0.931–1.104)	0.7502	–							0.4754
GP23	3	1.022(0.895–1.167)	0.7487	1.058(0.976–1.147)	0.1705	1.093(1.018–1.173)	0.0135	0.026(0.003–2.425)	0.1793	0.1782	0.0016

*MR* Mendelian randomization, *IVW* inverse variance weighting, *WM* weighted median, *PWM* penalized weighted median, *OR* odds ratio, *CI* confidence interval, *SNP* single-nucleotide polymorphism, *GP* glycan peak, *P*_heterogeneity_ is the *P*-value of Cochrane’s *Q* value in heterogeneity test by performing Inverse variance weighted method; *P*_pleiotropy_ is the *P*-value of MR-Egger intercept

### Associations of IgG N-glycans with inflammation

There was a suggestive association between genetically predicted higher GP14 and CRP in IVW models after multiple testing (OR, 1.009 for CRP per 1-SD higher in GP14, 95% CI = 1.001–1.032, *P* = 0.0350; Additional file 2: Table S5). In addition, as shown in Additional file 2: Table S6, genetically predicted IgG N-glycans were not associated with fibrinogen (for GP1-GP24, all *P*s > 0.05). The MR-Egger and Cochran’s Q method detected few evidences of directional pleiotropy and heterogeneity among each GP for CRP and fibrinogen (Additional file 2: Table S5 and Table S6), and IVW multiplicative random-effects models were used when heterogeneity existed. In the European population, there were suggestive associations between genetically determined IgG N-glycans on CRP after multiple testing (IVW OR, 1.031 for CRP per 1-SD higher GP12, 95% CI = 1.003–1.060, *P* = 0.0278; 1.032 for CRP per 1-SD higher GP13, 95% CI = 1.003–1.061, *P* = 0.0278; 1.066 for CRP per 1-SD higher GP22, 95% CI = 1.005–1.131, *P* = 0.0323; Additional file 2: Table S7). No directional pleiotropy was detected in MR-Egger method **(**Additional file 2: Table S7).

## Discussion

In the present study, we are the first to describe the associations of genetically predicted IgG N-glycans with T2D, and assess the potential effects of IgG N-glycans on inflammation (including CRP and fibrinogen) using two-sample MR study in East Asian and European populations, respectively. The MR results showed that increased genetically predicted IgG N-glycans of GP5 and GP13 were associated with a higher risk of T2D in the East Asian population, and genetically predicted decreased GP9 was associated with a higher risk of T2D in the European population. In addition, there were suggestive associations of genetically predicted IgG N-glycans on CRP both in East Asian and European populations.

Several previous observational studies have investigated that the associations between IgG N-glycans and risk of T2D (Lemmers et al. 2017; Li et al. 2019), whereas those results might be limited because of reverse causality or unmeasured confounding factors. Our analyses help to clarify the causal relationship and infer a direction of effect, which is not possible in observational studies due to potential reverse causation. In addition, our findings were consistent with previous studies that have also reported that N-glycosylation was associated with increased risk of T2D (Keser et al. 2017; Dotz et al. 2018; Rudman et al. 2019) and its complications (Itoh et al. 2007; Testa et al. 2015; Singh et al. 2020). A study involving three populations also confirmed that the increased complexity of plasma N-glycans including branching, galactosylation and sialylation are associated with higher risk of T2D (Keser et al. 2017). A case-cohort study presented that plasma N-glycome can identify high risk individuals before the onset of T2D, with the AUC value of the model consisting of plasma N-glycans to distinguish T2D from control as 0.83 (Wittenbecher et al. 2020).

Although the results of IgG N-glycans and risk of T2D were statistically significant, these findings were inconsistent in East Asian and European populations. The differences of associations between IgG N-glycans and T2D can be explained by ethnic differences of IgG N-glycans, as reported in previous studies (Gebrehiwot et al. 2018; Štambuk et al. 2020). IgG Fc segment is connected to complex-type biantennary N-glycan structures, which can control the binding affinity of IgG to activate or inhibit IgG Fcγ receptors, thereby affecting the effector function of IgG (Maverakis et al. 2015). The pro- and anti-inflammatory effects of IgG are modulated by modifications of N-glycan including galactose, fucose, sialic acid, and bisecting GlcNAc (Maverakis et al. 2015). In the East Asian population, our analyses showed that genetically determined GP5 and GP13 were increasingly associated with T2D. Specifically the results demonstrated that the pathophysiology of T2D were predominantly explained with increased of GP5 (abundance of a high-mannose N-glycan structure), as well as GP13 containing bisecting GlcNAc. In general, IgG carrying bisecting GlcNAc is associated with increased FcγRIII affinities, thus enhancing antibody-dependent cellular cytotoxicity (ADCC) activity (Zou et al. 2011). In consistent with our results, evidence from several studies showed that higher bisecting GlcNAc was elevated in response to inflammatory diseases (Vučković et al. 2015; Greto et al. 2021). However, effect of mannose N-glycan structure has not yet been fully clarified. Higher mannose GP5 was associated with systemic lupus erythematosus (Vučković et al. 2015), while previous studies showed that lower GP5 was associated with ischemic stroke (Liu et al. 2018b) and parkinson's disease (Russell et al. 2017). In the European population, genetically determined decreased GP9 was associated with T2D, suggesting that decreased galactosylation may increase the risk of T2D, which is consistent with previous studies (Nikolac Perkovic et al. 2014; Liu et al. 2018b). IgG N-glycans with decreased galactosylation mediate pro-inflammatory activity by recognizing mannose binding lectin (MBL) and activating subsequent complement (Matsumoto et al. 2000). Decreased galactosylation also enhance FcγRIII affinity, thus enhancing ADCC activity (Ackerman et al. 2013), and galactose deficiency also affects sialylation to regulate immune response.

The MR results provided only suggestive evidence that the effects of genetically determined IgG N-glycans on CRP, thus mediation analyses were technically unnecessary to perform. It is well know that T2D is a multifactorial disorder and a multi-layered process, besides regulating the immune response pathway, N-glycans is also involved in the etiology of insulin resistance (Pradhan et al. 2001). And inhibitory IgG receptor FcγRIIB plays an important role in activating obesity induced insulin resistance in microvascular endothelium (Tanigaki et al. 2018). Previous studies indicated that the faintly aberrant IgG glycosylation might play a cascading role in the pathogenesis of inflammatory disease (Liu et al. 2018b). A possible explanation for the inconsistent results in observational and our MR studies is that the sample sizes of IgG N-glycan GWAS from both European and Asian ethnic were too small to identify differences.

Although our study is a first MR analysis to explore this causality in East Asian and European populations so far, there are several limitations. Firstly, due to the relatively small sample sizes of East Asian ancestry, we did not filter out enough instrumental variables at *P* < 5 × 10^–8^. Therefore, we used SNPs meeting a more relaxed threshold at *P* < 1 × 10^–5^, which has been used in previous MR studies (Dong et al. 2021). The *F*-statistics of all selected SNPs are > 10, suggesting that all SNPs have sufficiently strong effects as IVs. Secondly, we only assessed CRP and fibrinogen as inflammation, but the levels of these inflammatory markers may not reflect the degree of inflammation in individuals. Thirdly, the effect sizes identified in our study are relatively small, however, the primary purpose of MR analysis is estimating the causal effects of exposures on outcome. Finally, since most SNPs related with IgG N-glycans could not be found corresponding SNPs in outcome of fibrinogen GWAS, IgG N-glycans and fibrinogen were not analyzed in the European population. This may also suggest that there is no causal relationship between genetically determined IgG N-glycan and fibrinogen.

## Conclusions

In conclusion, we demonstrated that genetically predicted IgG N-glycans were associated with T2D both in East Asian and European populations. In addition, there was suggestive genetic evidence for potential associations of genetically predicted IgG N-glycans and CRP. A more large-scale IgG N-glycan-QTL and further investigation are likely needed to validate our findings, and to clarify the mechanistic pathways of T2D.

## Supplementary Information

**Additional file 1: Table S2.** Results of each genetic variants (less than 3 SNPs) in the PhenoScanner v2. **Table S3.** Genetic variants used to instrument GPs in East Asian population. **Table S4.** Genetic variants used to instrument GPs in European population.

**Additional file 2: Table S1.** Description of glycans structures of 24 glycan peaks. **Table S6.** Mendelian randomization (MR) estimates of IgG glycans with fibrinogen from different MR methods in East Asian population. **Table S7.** Mendelian randomization (MR) estimates of IgG glycans with CRP from different MR methods in European population.

## Data Availability

The datasets supporting the conclusions of this article are available in the repositories listed in Table 1 and additional files.

## Abbreviations

T2D: Type 2 diabetes
IgG: Immunoglobulin G
MR: Mendelian randomization
CRP: C-reactive protein
IgG N-glycan-QTL: IgG N-glycan quantitative trait loci
GWAS: Genome-wide association study
SNP: Single nucleotide polymorphism
GP: Glycan peak
UPLC: Ultra-performance liquid chromatography
LD: Linkage disequilibrium
IV: Instrumental variable
IVW: Inverse variance weighting
WM: Weighted median
PWM: Penalized weighted median
CI: Confidence interval
ADCC: Antibody-dependent cellular cytotoxicity
MBL: Mannose binding lectin
