# Supplementary material for: Immunoglobulin G N-glycan, inflammation and type 2 diabetes in East Asian and European populations: a Mendelian randomization study
Source: Mol Med. 2022 Sep 14;28:114. doi: 10.1186/s10020-022-00543-z (PMC9476573; doi:10.1186/s10020-022-00543-z)
Supplement: Supplementary file 2 — Additional file 2: Table S1. Description of glycans structures of 24 glycan peaks. Table S6. Mendelian randomization (MR) estimates of IgG glycans with fibrinogen from different MR methods in East Asian population. Table S7. Mendelian randomization (MR) estimates of IgG glycans with CRP from different MR methods in European population. [file 10020_2022_543_MOESM2_ESM.docx]

Table S1. Description of glycans structures of 24 glycan peaks.

| Glycan peak | Structures | Graphic peak composition | Glycan peak | Structures | Graphic peak composition |
| --- | --- | --- | --- | --- | --- |
| GP1 | FA1 |  | GP13 | A2BG2 | 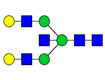 |
| GP2 | A2 |  | GP14 | FA2G2 | 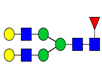 |
| GP3 | A2B |  | GP15 | FA2BG2 | 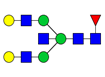 |
| GP4 | FA2 |  | GP16 | FA2G1S1 | 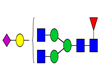 |
| GP5 | M5 | 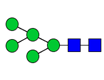 | GP17 | A2G2S1 | 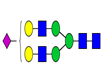 |
| GP6 | FA2B | 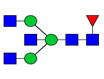 | GP18 | FA2G2S1 | 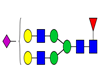 |
| GP7 | A2G1 | 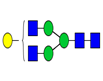 | GP19 | FA2BG2S1 | 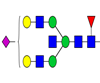 |
| GP8 | FA2[6]G1 | 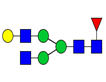 | GP20 | FA2FG2S1 |  |
| GP9 | FA2[3]G1 | 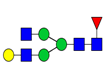 | GP21 | A2G2S2 | 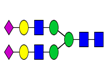 |
| GP10 | FA2[6]BG1 | 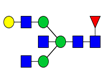 | GP22 | A2BG2S2 | 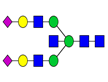 |
| GP11 | FA2[3]BG1 | 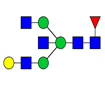 | GP23 | FA2G2S2 | 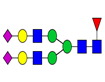 |
| GP12 | A2G2 | 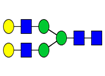 | GP24 | FA2BG2S2 | 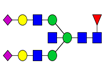 |

*GP* glycan peaks; *F* represents a fucose attached to core N-acetylglucosamine; *M* represents mannoses attached to core N-acetylglucosamine; *A* represents N-acetylglucosamine attached to core triple mannose; *B* represents bisected N-acetylglucosamine linked to core triple mannose; *G* represents galactose; *[3]G1* and *[6]G1* represent galactose attached to α1-3 and α1-6 mannose respectively; *S* represents N-acetylneuraminic acid attached to galactose; blue square represents N-acetylglucosamine; red triangle represents fucose; green circle represents mannose; yellow circle represents galactose; purple square represents N-acetylneuraminic acid.

Table S5. Mendelian randomization (MR) estimates of IgG glycans with CRP from different MR methods in East Asian population.

| Exposure | SNPs | IVW | |  | WM | |  | PWM | |  | MR-Egger | |  | *P*_pleiotropy_ | *P*_heterogeneity_ |
| --- | --- | --- | --- | --- | --- | --- | --- | --- | --- | --- | --- | --- | --- | --- | --- |
|  |  | OR (95%CI) | *P* | | OR (95%CI) | *P* | | OR (95%CI) | *P* | | OR (95%CI) | *P* | |  |  |
| GP1 | 33 | 1.001(0.994-1.007) | 0.800 | | 1.000(0.993-1.008) | 0.947 | | 0.999(0.991-1.007) | 0.779 | | 0.996(0.984-1.008) | 0.545 | | 0.383 | 0.004 |
| GP2 | 10 | 1.003(0.991-1.016) | 0.626 | | 1.008(0.991-1.026) | 0.347 | | 1.008(0.992-1.025) | 0.324 | | 1.012(0.984-1.041) | 0.429 | | 0.510 | 0.486 |
| GP3 | 17 | 0.999(0.990-1.008) | 0.809 | | 1.000(0.989-1.012) | 0.985 | | 1.000(0.989-1.012) | 0.959 | | 1.006(0.985-1.027) | 0.610 | | 0.504 | 0.400 |
| GP4 | 14 | 0.998(0.987-1.010) | 0.768 | | 1.004(0.989-1.018) | 0.618 | | 1.004(0.989-1.018) | 0.614 | | 0.981(0.961-1.002) | 0.099 | | 0.083 | 0.174 |
| GP5 | 20 | 1.001(0.992-1.009) | 0.908 | | 0.995(0.985-1.006) | 0.341 | | 0.994(0.984-1.005) | 0.244 | | 0.977(0.962-0.993) | 0.011 | | 0.004 | 0.096 |
| GP6 | 10 | 1.001(0.987-1.014) | 0.943 | | 1.008(0.990-1.026) | 0.410 | | 1.008(0.990-1.025) | 0.435 | | 0.999(0.971-1.027) | 0.920 | | 0.876 | 0.780 |
| GP7 | 11 | 0.998(0.989-1.007) | 0.617 | | 0.998(0.985-1.010) | 0.692 | | 0.998(0.985-1.010) | 0.705 | | 0.994(0.975-1.014) | 0.569 | | 0.697 | 0.778 |
| GP8 | 7 | 0.990(0.976-1.005) | 0.188 | | 0.994(0.975-1.015) | 0.587 | | 0.994(0.975-1.015) | 0.606 | | 0.950(0.886-1.019) | 0.209 | | 0.286 | 0.442 |
| GP9 | 8 | 0.997(0.985-1.009) | 0.615 | | 0.998(0.983-1.014) | 0.835 | | 0.998(0.982-1.015) | 0.841 | | 0.996(0.967-1.027) | 0.811 | | 0.963 | 0.843 |
| GP10 | 14 | 0.999(0.989-1.009) | 0.856 | | 1.003(0.989-1.016) | 0.728 | | 1.003(0.988-1.017) | 0.713 | | 1.002(0.977-1.028) | 0.878 | | 0.807 | 0.552 |
| GP11 | 6 | 0.996(0.984-1.009) | 0.565 | | 0.995(0.980-1.011) | 0.542 | | 0.995(0.980-1.011) | 0.553 | | 0.987(0.960-1.013) | 0.381 | | 0.464 | 0.562 |
| GP12 | 16 | 1.003(0.993-1.013) | 0.597 | | 1.005(0.992-1.018) | 0.435 | | 1.006(0.994-1.018) | 0.309 | | 1.013(0.992-1.035) | 0.258 | | 0.310 | 0.129 |
| GP13 | 11 | 0.996(0.987-1.006) | 0.448 | | 0.995(0.982-1.009) | 0.462 | | 0.995(0.983-1.008) | 0.464 | | 0.988(0.971-1.005) | 0.202 | | 0.280 | 0.687 |
| GP14 | 9 | 1.017(1.001-1.032) | 0.035 | | 1.016(0.995-1.038) | 0.137 | | 1.016(0.996-1.038) | 0.130 | | 1.050(1.010-1.091) | 0.043 | | 0.119 | 0.509 |
| GP15 | 9 | 0.998(0.982-1.015) | 0.842 | | 1.003(0.986-1.021) | 0.705 | | 1.003(0.986-1.021) | 0.707 | | 0.995(0.947-1.047) | 0.862 | | 0.905 | 0.081 |
| GP16 | 13 | 0.996(0.985-1.007) | 0.480 | | 0.999(0.986-1.013) | 0.916 | | 1.000(0.986-1.013) | 0.957 | | 1.020(0.991-1.052) | 0.226 | | 0.129 | 0.245 |
| GP17 | 20 | 1.007(0.999-1.015) | 0.088 | | 1.004(0.992-1.015) | 0.551 | | 1.004(0.992-1.015) | 0.523 | | 1.003(0.987-1.020) | 0.703 | | 0.631 | 0.463 |
| GP18 | 5 | 0.986(0.968-1.005) | 0.138 | | 0.983(0.961-1.006) | 0.147 | | 0.983(0.961-1.006) | 0.168 | | 0.960(0.896-1.029) | 0.337 | | 0.496 | 0.708 |
| GP19 | 13 | 0.998(0.988-1.008) | 0.634 | | 0.997(0.984-1.010) | 0.635 | | 0.997(0.966-1.029) | 0.639 | | 0.997(0.966-1.029) | 0.838 | | 0.951 | 0.775 |
| GP20 | 20 | 1.001(0.993-1.008) | 0.876 | | 1.002(0.991-1.013) | 0.716 | | 1.002(0.991-1.013) | 0.728 | | 0.999(0.979-1.020) | 0.960 | | 0.909 | 0.274 |
| GP21 | 8 | 1.010(0.998-1.022) | 0.111 | | 1.010(0.994-1.026) | 0.219 | | 1.010(0.993-1.028) | 0.206 | | 1.026(0.994-1.059) | 0.159 | | 0.325 | 0.820 |
| GP22 | 51 | 0.999(0.994-1.004) | 0.615 | | 0.997(0.991-1.003) | 0.272 | | 0.997(0.991-1.003) | 0.252 | | 1.003(0.993-1.013) | 0.583 | | 0.370 | 0.032 |
| GP23 | 8 | 0.998(0.986-1.010) | 0.715 | | 0.996(0.980-1.012) | 0.599 | | 0.996(0.980-1.011) | 0.597 | | 0.982(0.943-1.022) | 0.408 | | 0.445 | 0.696 |
| GP24 | 13 | 0.991(0.980-1.002) | 0.121 | | 0.985(0.971-1.000) | 0.042 | | 0.985(0.970-1.000) | 0.038 | | 0.977(0.933-1.022) | 0.333 | | 0.526 | 0.283 |

MR Mendelian randomization; CRP C-reactive protein; IVW inverse variance weighting; WM weighted median; PWM penalized weighted median; OR odds ratio; CI confidence interval; SNP single-nucleotide polymorphism; GP, glycan peak; *P*_heterogeneity_ is the *P*-value of Cochrane’s *Q* value in heterogeneity test by performing Inverse variance weighted method; *P*_pleiotropy_ is the *P*-value of MR-Egger intercept.

Table S6. Mendelian randomization (MR) estimates of IgG glycans with fibrinogen from different MR methods in East Asian population.

| Exposure | SNPs | IVW | |  | WM | |  | PWM | |  | MR-Egger | |  | *P*_pleiotropy_ | *P*_heterogeneity_ |
| --- | --- | --- | --- | --- | --- | --- | --- | --- | --- | --- | --- | --- | --- | --- | --- |
|  |  | OR (95%CI) | *P* | | OR (95%CI) | *P* | | OR (95%CI) | *P* | | OR (95%CI) | *P* | |  |  |
| GP1 | 33 | 0.992(0.981-1.003) | 0.170 | | 0.999(0.982-1.016) | 0.887 | | 0.999(0.982-1.015) | 0.928 | | 0.992(0.971-1.014) | 0.501 | | 0.960 | 0.083 |
| GP2 | 10 | 0.999(0.974-1.024) | 0.932 | | 1.006(0.970-1.043) | 0.748 | | 1.006(0.971-1.042) | 0.740 | | 1.053(0.994-1.115) | 0.115 | | 0.080 | 0.415 |
| GP3 | 17 | 1.006(0.988-1.024) | 0.510 | | 0.999(0.974-1.024) | 0.938 | | 0.999(0.974-1.025) | 0.939 | | 0.989(0.948-1.033) | 0.632 | | 0.416 | 0.407 |
| GP4 | 14 | 0.998(0.977-1.020) | 0.894 | | 1.002(0.975-1.031) | 0.861 | | 1.002(0.974-1.032) | 0.867 | | 1.023(0.982-1.065) | 0.292 | | 0.196 | 0.287 |
| GP5 | 20 | 1.001(0.986-1.015) | 0.924 | | 1.000(0.980-1.021) | 0.999 | | 1.000(0.980-1.021) | 0.997 | | 1.013(0.981-1.046) | 0.437 | | 0.411 | 0.482 |
| GP6 | 10 | 1.010(0.979-1.042) | 0.532 | | 1.022(0.983-1.063) | 0.273 | | 1.025(0.986-1.067) | 0.210 | | 0.965(0.913-1.020) | 0.239 | | 0.098 | 0.237 |
| GP7 | 11 | 0.989(0.968-1.011) | 0.333 | | 0.983(0.956-1.010) | 0.210 | | 0.981(0.955-1.009) | 0.186 | | 0.999(0.953-1.047) | 0.966 | | 0.662 | 0.193 |
| GP8 | 7 | 1.004(0.971-1.038) | 0.817 | | 1.014(0.973-1.058) | 0.504 | | 1.014(0.973-1.057) | 0.500 | | 0.986(0.829-1.172) | 0.877 | | 0.841 | 0.264 |
| GP9 | 8 | 0.989(0.961-1.018) | 0.447 | | 0.986(0.949-1.025) | 0.477 | | 0.986(0.950-1.024) | 0.468 | | 0.999(0.927-1.077) | 0.977 | | 0.783 | 0.232 |
| GP10 | 14 | 1.008(0.988-1.030) | 0.430 | | 1.005(0.978-1.032) | 0.741 | | 1.005(0.979-1.030) | 0.726 | | 1.036(0.984-1.091) | 0.204 | | 0.283 | 0.899 |
| GP11 | 6 | 1.003(0.977-1.030) | 0.807 | | 0.995(0.964-1.028) | 0.782 | | 0.995(0.964-1.028) | 0.781 | | 0.974(0.921-1.031) | 0.416 | | 0.314 | 0.717 |
| GP12 | 16 | 0.997(0.979-1.015) | 0.720 | | 1.006(0.981-1.032) | 0.631 | | 1.006(0.982-1.031) | 0.619 | | 0.977(0.942-1.014) | 0.238 | | 0.249 | 0.373 |
| GP13 | 11 | 0.988(0.969-1.008) | 0.250 | | 0.993(0.967-1.019) | 0.582 | | 0.993(0.968-1.018) | 0.575 | | 1.005(0.968-1.043) | 0.813 | | 0.338 | 0.341 |
| GP14 | 9 | 1.025(0.980-1.072) | 0.290 | | 1.024(0.977-1.073) | 0.328 | | 1.024(0.978-1.071) | 0.309 | | 1.044(0.928-1.176) | 0.495 | | 0.738 | 0.032 |
| GP15 | 9 | 1.019(0.989-1.050) | 0.222 | | 1.032(0.997-1.068) | 0.073 | | 1.039(1.004-1.075) | 0.029 | | 1.054(0.967-1.150) | 0.269 | | 0.433 | 0.176 |
| GP16 | 13 | 1.007(0.987-1.027) | 0.504 | | 1.015(0.989-1.043) | 0.256 | | 1.015(0.988-1.043) | 0.268 | | 1.004(0.946-1.065) | 0.904 | | 0.913 | 0.893 |
| GP17 | 20 | 0.998(0.981-1.016) | 0.850 | | 0.989(0.967-1.011) | 0.323 | | 0.987(0.964-1.010) | 0.271 | | 1.024(0.989-1.059) | 0.199 | | 0.120 | 0.237 |
| GP18 | 5 | 0.968(0.932-1.005) | 0.090 | | 0.971(0.923-1.021) | 0.253 | | 0.971(0.927-1.018) | 0.219 | | 0.939(0.815-1.082) | 0.448 | | 0.694 | 0.702 |
| GP19 | 13 | 0.989(0.965-1.014) | 0.393 | | 0.999(0.970-1.029) | 0.953 | | 0.996(0.967-1.026) | 0.772 | | 0.963(0.889-1.043) | 0.372 | | 0.497 | 0.121 |
| GP20 | 20 | 0.987(0.973-1.001) | 0.072 | | 0.987(0.968-1.006) | 0.176 | | 0.987(0.967-1.006) | 0.182 | | 0.996(0.959-1.035) | 0.854 | | 0.605 | 0.765 |
| GP21 | 8 | 0.999(0.975-1.024) | 0.928 | | 0.996(0.965-1.029) | 0.826 | | 0.996(0.965-1.029) | 0.829 | | 1.003(0.941-1.070) | 0.930 | | 0.896 | 0.514 |
| GP22 | 51 | 1.002(0.994-1.011) | 0.561 | | 1.000(0.988-1.012) | 0.999 | | 1.000(0.988-1.012) | 0.999 | | 1.000(0.983-1.017) | 0.988 | | 0.746 | 0.823 |
| GP23 | 8 | 0.997(0.973-1.022) | 0.824 | | 0.998(0.967-1.031) | 0.916 | | 0.998(0.967-1.031) | 0.916 | | 0.993(0.908-1.086) | 0.882 | | 0.925 | 0.395 |
| GP24 | 13 | 0.994(0.974-1.015) | 0.567 | | 0.997(0.969-1.026) | 0.840 | | 0.997(0.970-1.025) | 0.834 | | 1.011(0.930-1.098) | 0.800 | | 0.693 | 0.698 |

MR Mendelian randomization; IVW inverse variance weighting; WM weighted median; PWM penalized weighted median; OR odds ratio; CI confidence interval; SNP single-nucleotide polymorphism; GP, glycan peak; *P*_heterogeneity_ is the *P*-value of Cochrane’s *Q* value in heterogeneity test by performing Inverse variance weighted method; *P*_pleiotropy_ is the *P*-value of MR-Egger intercept.

Table S7. Mendelian randomization (MR) estimates of IgG glycans with CRP from different MR methods in European population.

| Exposure | SNPs | IVW | |  | WM | |  | PWM | |  | MR-Egger | |  | *P*_pleiotropy_ | *P*_heterogeneity_ |
| --- | --- | --- | --- | --- | --- | --- | --- | --- | --- | --- | --- | --- | --- | --- | --- |
|  |  | OR (95%CI) | *P* | | OR (95%CI) | *P* | | OR (95%CI) | *P* | | OR (95%CI) | *P* | |  |  |
| GP2 | 7 | 1.019(0.995-1.046) | 0.124 | | 1.028(1.007-1.049) | 0.007 | | 1.030(1.009-1.051) | 0.005 | | 1.009(0.935-1.089) | 0.817 | | 0.789 | 0.015 |
| GP4 | 2 | 0.988(0.945-1.032) | 0.593 | | - | | | | | | | | | | 0.088 |
| GP6 | 4 | 0.982(0.931-1.036) | 0.508 | | 0.969(0.931-1.008) | 0.116 | | 0.955(0.915-0.998) | 0.041 | | 0.911(0.539-1.539) | 0.761 | | 0.012 | 0.036 |
| GP7 | 5 | 1.022(0.981-1.065) | 0.288 | | 1.033(1.003-1.064) | 0.029 | | 1.035(1.005-1.065) | 0.018 | | 1.010(0.903-1.131) | 0.869 | | 0.835 | 0.010 |
| GP8 | 2 | 0.999(0.954-1.045) | 0.954 | | - | | | | | | | | | | 0.759 |
| GP9 | 4 | 0.998(0.961-1.038) | 0.940 | | 0.992(0.962-1.024) | 0.660 | | 0.987(0.957-1.019) | 0.446 | | 1.040(0.874-1.238) | 0.699 | | 0.679 | 0.064 |
| GP10 | 5 | 0.998(0.960-1.037) | 0.942 | | 0.995(0.966-1.025) | 0.774 | | 0.994(0.966-1.024) | 0.736 | | 0.999(0.878-1.136) | 0.992 | | 0.992 | 0.003 |
| GP11 | 3 | 1.001(0.952-1.051) | 0.966 | | 0.996(0.960-1.033) | 0.845 | | 0.996(0.960-1.033) | 0.843 | | 0.931(0.772-1.122) | 0.592 | | 0.573 | 0.055 |
| GP12 | 2 | 1.031(1.003-1.060) | 0.028 | | - | | | | | | | | | | 0.748 |
| GP13 | 2 | 1.032(1.003-1.061) | 0.028 | | - | | | | | | | | | | 0.748 |
| GP14 | 4 | 1.019(0.994-1.046) | 0.133 | | 1.013(0.982-1.044) | 0.403 | | 1.013(0.981-1.046) | 0.427 | | 1.043(0.937-1.161) | 0.517 | | 0.708 | 0.749 |
| GP15 | 6 | 0.998(0.957-1.042) | 0.954 | | 1.002(0.957-1.042) | 0.875 | | 1.004(0.967-1.042) | 0.824 | | 1.086(0.823-1.433) | 0.590 | | 0.580 | 0.005 |
| GP16 | 5 | 0.996(0.972-1.020) | 0.752 | | 0.989(0.976-1.003) | 0.142 | | 0.988(0.976-1.001) | 0.079 | | 0.951(0.902-1.003) | 0.165 | | 0.168 | 0.002 |
| GP18 | 3 | 0.980(0.945-1.015) | 0.272 | | 0.977(0.949-1006) | 0.134 | | 0.977(0.950-1.006) | 0.129 | | 1.125(0.965-1.312) | 0.372 | | 0.325 | 0.123 |
| GP20 | 2 | 0.949(0.763-1.178) | 0.633 | | - | | | | | | | | | | <0.001 |
| GP22 | 2 | 1.066(1.005-1.131) | 0.032 | | - | | | | | | | | | | 0.033 |
| GP23 | 3 | 0.973(0.941-1.006) | 0.113 | | 0.959(0.927-0.994) | 0.022 | | 0.958(0.927-0.990) | 0.011 | | 0.642(0.121-3.388) | 0.710 | | 0.183 | 0.002 |

MR Mendelian randomization; IVW inverse variance weighting; WM weighted median; PWM penalized weighted median; OR odds ratio; CI confidence interval; SNP single-nucleotide polymorphism; GP, glycan peak; *P*_heterogeneity_ is the *P*-value of Cochrane’s *Q* value in heterogeneity test by performing Inverse variance weighted method; *P*_pleiotropy_ is the *P*-value of MR-Egger intercept.
